# Hydrashift 2/4 daratumumab检测消除达雷妥尤单抗对血清免疫固定电泳干扰的应用

**DOI:** 10.3760/cma.j.issn.0253-2727.2021.10.008

**Published:** 2021-10

**Authors:** 双 徐, 扬 刘, 磊 温, 磊 赵, 旭 邓, 嵘 荣, 瑾 路

**Affiliations:** 1 北京大学人民医院检验科，北京 100044 Department of Clinical Laboratory, Peking University People's Hospital, Beijing 100044, China; 2 北京大学人民医院血液科，北京大学血液病研究所，国家血液系统疾病临床医学研究中心，北京 100044 Department of Hematology, Peking University People's Hospital, Peking University Institute of Hematology, National Clinical Research Center for Hematologic Disease, Beijing 100044, China; 3 血液学协同创新中心，苏州 215006 Center for Collaborative Innovation in Hematology, Suzhou 215006, China

**Keywords:** 达雷妥尤单抗, 免疫固定电泳, 多发性骨髓瘤, Daratumumab, Immunofixation electrophoresis, Multiple myeloma

## Abstract

**目的:**

探讨达雷妥尤单抗治疗浆细胞疾病后对免疫固定电泳结果产生的干扰及消除干扰的方法。

**方法:**

收集2020年4月至2021年3月北京大学人民医院应用达雷妥尤单抗治疗的8例浆细胞疾病患者的血清样本，分别进行标准的免疫固定电泳检测及Hydrashift 2/4 daratumumab检测。

**结果:**

达雷妥尤单抗治疗后，81.3％（13/16）的样本出现药物性单克隆条带（IgG-κ型），未出现药物性单克隆条带的样本考虑与个体差异、给药间隔时间、患者免疫球蛋白水平相关。出现IgG-κ型单克隆条带的样本中76.9％（10/13）可通过免疫固定电泳直接分辨为内源性或外源性单克隆条带，余3份样本（3/13）需要使用Hydrashift 2/4 daratumumab检测进行区分。

**结论:**

应用Hydrashift 2/4 daratumumab检测可去除达雷妥尤单抗对免疫固定电泳结果的干扰，辅助评判疗效。

## Abstract (en)

**Objective:**

To investigate the interference of daratumumab on immunofixation electrophoresis after treating plasma cell diseases and methods to eliminate the interference.

**Methods:**

Serum samples of eight patients with plasma cell diseases treated with daratumumab in Peking University People's Hospital from April 2020 to March 2021 were collected for standard immunofixation electrophoresis and Hydrashift 2/4 daratumumab assay.

**Results:**

After treatment, 81.3％ (13/16) of the samples showed drug-induced monoclonal antibodies (IgG-κ). The samples without drug-induced monoclonal bands were related to individual differences, administration intervals, and immunoglobulin levels. Among the samples with IgG-κ monoclonal bands, 76.9％ (10/13) could be directly identified as endogenous or exogenous monoclonal bands by immunofixation electrophoresis, and the others (3/13) could be identified by Hydrashift 2/4 daratumumab assay.

**Conclusion:**

Hydrashift 2/4 daratumumab assay can remove the band of daratumumab on the immunofixation electrophoresis and help with efficacy evaluation.

多发性骨髓瘤（MM）是一种恶性克隆性浆细胞异常增殖性疾病，中国人群发病的中位年龄为59岁[1]，发病率在血液系统肿瘤中位居第2位。随着蛋白酶体抑制剂以及免疫调节剂等新药在临床的应用，MM患者的预后得到显著改善。然而，几乎所有患者仍不可避免地出现疾病进展，并且对蛋白酶体抑制剂和免疫调节剂耐药。达雷妥尤单抗是一种完全人源性抗CD38单克隆抗体，在2015年被美国食品药品管理局（FDA）批准用于复发难治性MM的治疗[2]，2021年被批准用于初治系统性轻链型淀粉样变性（systemic light chain amyloidosis，AL）的治疗，2019年在中国获批用于复发MM的治疗。

由于达雷妥尤单抗为IgG-κ型单克隆抗体，因此在进行血清蛋白电泳（SPE）和血清免疫固定电泳（IFE）时可被检测为单克隆带，干扰疾病疗效判定[3]。Hydrashift 2/4 daratumumab分析可以用于区分内源性（疾病引起的）单克隆条带和外源性（药物性）单克隆条带，并于2019年被FDA批准应用于临床，但在中国尚无使用经验。本文展示了达雷妥尤单抗在不同类型浆细胞疾病患者样本中的IFE图形，以及使用Hydrashift 2/4 daratumumab试剂盒分析后药物性单克隆抗体在IFE中的迁移，是应用达雷妥尤单抗后使用Hydrashift 2/4 daratumumab检测的国内首次报道，为临床确定Hydrashift 2/4daratumumab的应用时机等提供帮助。

## 病例与方法

1. 病例：收集2020年4月至2021年3月北京大学人民医院应用以达雷妥尤单抗为主方案治疗的8例浆细胞疾病患者的血清样本。每例患者预计采集三个时间点的样本进行检测：T0（单抗治疗前）、T1（第2个疗程第1天）、T2（第3个疗程第1天），共收集8例患者的24份血清样本。本研究经北京大学人民医院伦理委员会批准（批准文号：2017PHB005-01）。

2. 仪器与试剂：患者的样本分别进行标准的IFE检测及Hydrashift 2/4 daratumumab检测。两者均使用法国Sebia公司全自动琼脂糖凝胶电泳仪（Sebia Hydrasys 2电泳仪）、配套5种抗血清［γ重链（IgG）、α重链（IgA）、µ重链（IgM）、抗κ轻链（游离和非游离）、抗λ轻链（游离和非游离）］及4人份琼脂糖凝胶片进行检测。

Hydrashift 2/4 daratumumab检测分析试剂盒为法国Sebia公司生产，主要包括抗达雷妥尤单抗抗体（anti-daratumumab）、加样器及稀释液等。其分析过程与正常的IFE分析过程类似，仅需增加一个步骤：将加入抗达雷妥尤单抗抗体的加样器在琼脂糖凝胶板上点样，继而抗达雷妥尤单抗抗体特异性结合达雷妥尤单抗，生成达雷妥尤单抗/抗达雷妥尤单抗抗体络合物，并迁移到γ球蛋白区带之外。

3. 结果判读：全部IFE结果均由2名经验丰富的技术人员同时判断。

4. 疗效判定：MM疗效评估根据《中国多发性骨髓瘤诊治指南（2020年修订）》的标准[4]。AL疗效评估根据《系统性轻链型淀粉样变性诊断和治疗指南》中的标准[5]。

## 结果

1. 患者基本信息及达雷妥尤单抗治疗后血清IFE典型改变：8例患者在使用达雷妥尤单抗治疗前，IFE中均出现内源性单克隆抗体条带，其中IgG-κ型1例，IgG-λ型3例，IgA-κ型1例，IgD-λ型1例，轻链κ型2例。使用达雷妥尤单抗治疗后，7例患者样本可在T1观察到IgG-κ型单克隆抗体条带，6例患者样本在T2出现IgG-κ条带（表1）。例1应用达雷妥尤单抗前后的血清IFE结果见图1。

**表1:** 8例浆细胞疾病患者的基本信息及达雷妥尤单抗（Dara）治疗后免疫固定电泳结果

例号	性别	年龄（岁）	分型	治疗方案	Dara用药剂量(mg/kg)	T0		T1		
						单克隆条带类型	M蛋白含量	单克隆条带类型	M蛋白含量	综合疗效评估
1	男	57	IgA-κ型	Dara+BD	16	IgA-κ	SPE 4.5 g	IgA-κ，IgG-κ弱	SPE 0.3 g	VGPR
2	女	57	IgG-λ型	Dara+BD	16	IgG-λ	SPE 31.7 g	IgG-λ，IgG-κ弱	SPE 11.2 g	PR
3	男	77	轻链κ型，伴中枢浆细胞瘤	Dara+BD	16	轻链κ	SPE阴性；24 h尿κ轻链：11 865 mg	轻链κ弱	SPE阴性；24 h尿κ轻链：202 mg/L^a^	PR
4	男	59	IgG-λ型	Dara+BD	16	IgG-λ	SPE 21.2 g	IgG-λ，IgG-κ弱	SPE 12.1 g	MR
5	女	72	IgD-λ型，伴髓外浆细胞瘤	Dara+Rd	16	IgD-λ	SPE阴性；24 h尿λ轻链：2635 mg	IgD-λ弱，IgG-κ弱	SPE阴性；24 h尿λ轻链：<50 mg/L^a^；血游离轻链：κ：5.5 mg/L，λ：125 mg/L，κ/λ：0.044	SD（髓外浆细胞瘤无变化）
6	男	60	轻链κ型	Dara+BD	16	轻链κ弱	SPE阴性；24 h尿κ轻链：113 mg，血游离轻链：κ：1205 mg/L，λ：16.1 mg/L，κ/λ：74.8	IgG-κ弱	SPE阴性；24 h尿κ轻链：38 mg，血游离轻链：κ：655 mg/L，λ：12 mg/L，κ/λ：54.5	MR
7	男	60	IgG-λ型	Dara+BD	16	IgG-λ	SPE 5.7 g；血游离轻链：κ：20.1 mg/L，λ：84.7 mg/L，κ/λ：0.23	IgG-λ，IgG-κ弱	SPE 2.4g；血游离轻链：κ：8.91 mg/L，λ：12.2 mg/L，κ/λ：0.73	VGPR
8	男	56	IgG-κ型	Dara+DECP	16	IgG-κ	SPE 39.2 g	IgG-κ	SPE 32.8 g	SD

例号	性别	年龄（岁）	T2		
			单克隆条带类型	M蛋白含量	综合疗效评估
1	男	57	IgG-κ	SPE 0.7 g	第二克隆/CR？
2	女	57	IgG-λ，IgG-κ弱	SPE 3.0 g	VGPR
3	男	77	轻链κ弱	SPE阴性；24 h尿κ轻链：529 mg	PR
4	男	59	IgG-λ	SPE 5.3 g	PR
5	女	72	IgD-λ弱， IgG-κ弱	SPE阴性；24 h尿λ轻链：<50 mg/L^a^，血游离轻链：κ：5.5 mg/L， λ：140 mg/L，κ/λ：0.039	PR（髓外浆细胞瘤缩小> 50%）
6	男	60	IgG-κ弱	SPE阴性； 24 h尿κ轻链：36 mg，血游离轻链：κ：830 mg/L，λ：19.7 mg/L，κ/λ：42.1	MR
7	男	60	IgG-λ，IgG-κ弱	SPE 0.5 g；血游离轻链：κ：8.06 mg/L，λ：9.52 mg/L，κ/λ：0.847	VGPR
8	男	56	IgG-κ	SPE 32.7 g	SD

注：除例7诊断为系统性轻链型淀粉样变性外，余患者均为复发难治性多发性骨髓瘤患者。T0：单抗治疗前；T1：第2个疗程第1天；T2：第3个疗程第1天；BD：硼替佐米+地塞米松；Rd：来那度胺+地塞米松；DECP：顺铂+环磷酰胺+依托泊苷+地塞米松；SPE：血清蛋白电泳；VGPR：非常好的部分缓解；PR：部分缓解；MR：微小缓解；SD：疾病稳定；CR：完全缓解；a：缺少患者24 h尿量，因此用浓度表示

**图1:**
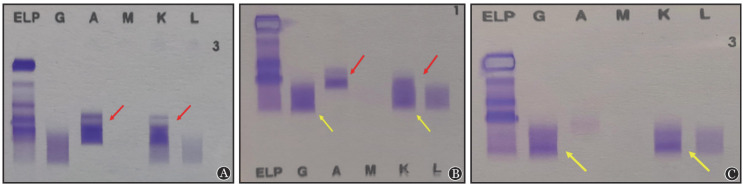
例1应用达雷妥尤单抗前后血清免疫固定电泳（IFE）结果 A：达雷妥尤单抗治疗前IFE结果，红箭头所示为内源性IgA-κ型单克隆条带；B：达雷妥尤单抗治疗第2个疗程第1天IFE结果，红箭头所示为内源性IgA-κ型单克隆条带，M蛋白较前减少，且在IgG、κ泳道出现较弱的IgG-κ单克隆条带（黄箭头所示）；C：达雷妥尤单抗治疗第3个疗程第1天IFE结果，患者IgA-κ型单克隆条带消失，出现较明显的IgG-κ单克隆条带（黄箭头所示），此时判断为第二克隆出现或完全缓解

2. 应用达雷妥尤单抗治疗后未检出外源性单克隆条带的血清IFE样本：单抗治疗后的16份样本中，3份样本未检出IgG-κ单克隆条带。例3为轻链κ型MM患者，经达雷妥尤单抗治疗后，其IFE结果中未检出IgG-κ单克隆条带。另1例为IgG-λ型MM患者（例4），T1可见IgG-κ单克隆条带，而T2无IgG-κ单克隆条带。

3. 使用Hydrashift 2/4 daratumumab检测后的样本血清IFE改变：13份可以观察到外源性IgG-κ单克隆条带的患者血清样本中，例1的T2样本仅存在IgG-κ单克隆条带，原有类型内源性单克隆条带消失，不能区分是治疗后免疫重建过程中出现的寡克隆带、第二克隆复发还是外源性单克隆条带。例6为轻链κ型MM患者，单抗治疗后，T1、T2样本只能观察到弱IgG-κ条带，不能分辨是内源性条带被外源性条带遮盖还是内源性条带消失；余10份样本的血清IFE均可分辨是内源性M蛋白还是外源性单克隆条带（表2）。在使用Hydrashift 2/4 daratumumab检测后，例1的T2样本IFE结果为阴性，此时疗效评估应为完全缓解（CR）。例6的T1及T2样本Hydrashift 2/4 daratumumab检测示IFE阴性，即无内源性单克隆条带（图2），但由于患者SPE阴性，24 h尿轻链<200 mg，因此需要使用血清游离轻链进行疗效评估，尽管血清IFE阴性，但总体的疗效评估仍为微小缓解。余10份样本进行Hydrashift 2/4daratumumab分析后所有外源性单克隆条带均可被迁移出γ区，例8应用达雷妥尤单抗前后的血清IFE结果见图3。

**表2:** 8例浆细胞疾病患者Hydrashift 2/4 daratumumab检测前后血清免疫固定电泳（IFE）结果

例号	T0	T1				T2			
	IFE结果	IFE结果	距末次应用达雷妥尤单抗时间	IFE能否区分内源性、外源性MG	加入抗达雷妥尤单抗试剂后IFE结果	IFE结果	距末次应用达雷妥尤单抗时间	IFE能否区分内源性、外源性MG	加入抗达雷妥尤单抗试剂后IFE结果
1	IgA-κ	IgA-κ，IgG-κ弱	14d	Y	IgA-κ	IgG-κ	20d	N	阴性
2	IgG-λ	IgG-λ，IgG-κ弱	14d	Y	IgG-λ	IgG-λ，IgG-κ弱	41d	Y	IgG-λ
3	游离κ	游离κ弱	24d	−	游离κ	游离κ	31d	−	游离κ
4	IgG-λ	IgG-λ，IgG-κ弱	14d	Y	IgG-λ	IgG-λ	37d	−	IgG-λ
5	IgD-λ	IgD-λ弱，IgG-κ弱	21d	Y	IgD-λ弱	IgD-λ弱，IgG-κ弱	54d	Y	IgD-λ弱
6	游离κ弱	IgG-κ弱	10d	N	阴性	IgG-κ弱	8d	N	阴性
7	IgG-λ	IgG-λ，IgG-κ弱	9d	Y	IgG-λ	IgG-λ，IgG-κ弱	9d	Y	IgG-λ
8	IgG-κ	IgG-κ	9d	Y	IgG-κ	IgG-κ	26d	Y	IgG-κ

注：T0：单抗治疗前；T1：第2个疗程第1天；T2：第3个疗程第1天；MG：单克隆免疫球蛋白；Y：IFE可以区分内源性或外源性MG；N：IFE结果不能区分内源性或外源性MG；−：未检出外源性单克隆条带

**图2:**
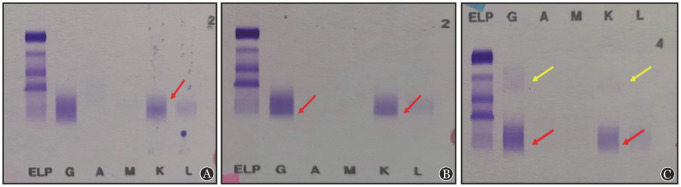
例6 Hydrashift 2/4 daratumumab检测前后血清免疫固定电泳（IFE）结果 A：治疗前患者IFE结果为游离κ型M蛋白（红箭头）；B：达雷妥尤单抗治疗第2个疗程第1天IFE结果，可见较弱IgG-κ单克隆条带（红箭头），此时不能辨别是否存在内源性M蛋白；C：加入抗达雷妥尤单抗试剂后可见γ区IgG-κ单克隆条带消失（红箭头），α区出现达雷妥尤单抗/抗达雷妥尤单抗抗体络合物弥散的印记（黄箭头），此患者第2个疗程第1天的IFE结果应为阴性，即无内源性M蛋白

**图3:**
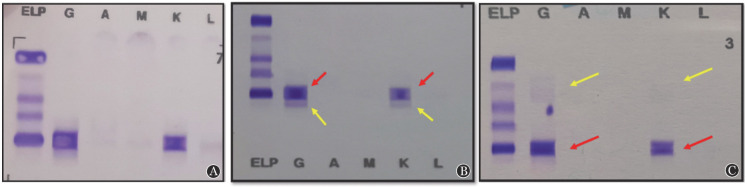
例8 Hydrashift 2/4 daratumumab检测前后血清免疫固定电泳（IFE）结果 A：治疗前患者IFE显示为IgG-κ型M蛋白；B：达雷妥尤单抗治疗第2个疗程第1天IFE结果，红箭头所示为患者内源性IgG-κ单克隆条带，黄箭头所示为药物性单克隆IgG-κ条带；C：达雷妥尤单抗治疗第2个疗程第1天的样本加入抗达雷妥尤单抗试剂后IFE结果，可见β区外源性单克隆条带消失，仅剩内源性M蛋白（红箭头），α区出现了达雷妥尤单抗/抗达雷妥尤单抗抗体络合物弥散的印记（黄箭头）

## 讨论

依据《中国多发性骨髓瘤诊治指南（2020年修订）》[4]，血清和尿IFE是判断MM及其他浆细胞疾病疗效的重要指标，其结果与判断疾病是否缓解、缓解深度及预后密切相关。如CR的判断标准包括血清和尿液IFE阴性，患者使用达雷妥尤单抗后，该药物作为一种单克隆免疫球蛋白可在SPE/IFE中被检测到，使原本被判定为CR的患者被评估为非常好的部分缓解。有研究报道，达雷妥尤单抗会在IFE上靠近阴极的γ区出现条带[6]，根据目前推荐的16 mg/kg给药方案，达雷妥尤单抗平均血清峰值浓度约为0.9 g/L，而IFE检测下限为0.2 g/L，该药物可能干扰IFE结果[7]–[8]。McCudden等[9]进行了达雷妥尤单抗浓度稀释梯度实验，在达雷妥尤单抗浓度大于0.25 g/L时能被检测到，我们未进行浓度稀释梯度实验。本研究结果显示，在使用推荐剂量的达雷妥尤单抗后，81.3％（13/16）的样本会出现药物性单克隆条带，进而影响IFE结果的判读，导致假阳性结果，使临床医师无法判断CR。

但并不是所有患者均会出现外源性IgG-κ条带。药物性单克隆条带能否在IFE上被观察到受三个因素影响：①患者输注的药物浓度；②患者免疫球蛋白水平，免疫球蛋白含量高会遮盖药物的条带；③内源性M蛋白条带位置与药物性单克隆条带重叠[9]。本研究中8例患者均使用达雷妥尤单抗标准剂量（16 mg/kg）输注，例3的T1、T2样本，例4的T2样本未检测到外源性单克隆条带，考虑药物性单克隆条带的出现还受个体药物代谢差异及给药时间间隔等多种因素的影响。Tang等[7]的研究显示，所有患者在每周输注期间均持续检测到药物性单克隆抗体条带，但在每两周或每月（维持）输注期间能否观察到外源性单克隆条带不确定。

Thoren等[10]的研究报道，40例样本中27例（5例IgG-κ型，2例游离κ型，20例其他类型）在IFE上可直接区分内源性及外源性单克隆条带，余13例样本（IgG-κ及游离κ型）因内源性与外源性单克隆条带在琼脂糖凝胶上有相同的迁移速度导致单克隆条带重合，在IFE上并不能区分。本研究出现外源性单克隆条带的样本中，76.9％（10/13）可通过IFE直接区分其为内源性还是外源性单克隆条带，余3份样本（3/13）需要使用Hydrashift 2/4 daratumumab检测进行区分。国外研究对含有达雷妥尤单抗的商业血清样本和使用达雷妥尤单抗治疗的患者样本均进行了达雷妥尤单抗移除实验，证明并验证了该实验的有效性[9],[11]。在我们的实验中达雷妥尤单抗均可被抗达雷妥尤单抗抗体迁移出γ区（13/13）。但我们的样本量较小，未观察到达雷妥尤单抗与内源性M蛋白有相同迁移速度导致单克隆条带重叠的现象，3份需进行达雷妥尤单抗移除实验的样本均为内源性条带消失，是被外源性单克隆条带干扰所致。达雷妥尤单抗对IFE结果的干扰主要存在于IgG-κ型及游离κ型患者，在本研究中IgG-κ型及游离κ型样本量偏少，我们将在后续实验中继续收集此型样本进行检测。

这种分析方法也有一定局限性，Hydrashift 2/4daratumumab分析仅针对达雷妥尤单抗，不与其他单克隆抗体反应。另一种单克隆抗体Elotuzumab也已经应用于临床治疗，如使用此药物或联合达雷妥尤单抗进行治疗，Hydrashift 2/4 daratumumab试剂则不能消除该药物引起的IFE干扰[9]。另外，Hydrashift 2/4 daratumumab分析仅能应用于IFE，定量M蛋白的SPE不能通过此方法解决。

总之，由于在浆细胞疾病治疗的过程中越来越多地使用单克隆抗体，因此在常规随访进行SPE、IFE检测时，临床医师应仔细辨别其结果是否受药物干扰，因考虑到受达雷妥尤单抗影响者主要为接受治疗的IgG-κ型及游离κ型患者，而血清中达雷妥尤单抗最高浓度为0.9 g/L，IFE检测下限为0.2 g/L，因此建议M蛋白含量在1.2 g/L以下的IgG-κ型及游离κ型的患者进行达雷妥尤单抗移除实验以除外药物影响，M蛋白大于1.2 g/L考虑存在内源性M蛋白，无需使用Hydrashift 2/4 daratumumab检测。当非IgG-κ型及游离κ型患者出现IgG-κ单克隆条带时，为与治疗后出现的寡克隆带或第二克隆区分，也可进行达雷妥尤单抗移除实验，以得到准确IFE结果。
